# State-dependent effective interactions in oscillator networks through coupling functions with dead zones

**DOI:** 10.1098/rsta.2019.0042

**Published:** 2019-10-28

**Authors:** Peter Ashwin, Christian Bick, Camille Poignard

**Affiliations:** EPSRC Centre for Predictive Modelling in Healthcare and Centre for Systems, Dynamics & Control, Department of Mathematics, University of Exeter, Exeter, Devon, UK

**Keywords:** coupled oscillator, network dynamical system, coupling function

## Abstract

The dynamics of networks of interacting dynamical systems depend on the nature of the coupling between individual units. We explore networks of oscillatory units with coupling functions that have ‘dead zones’, that is the coupling functions are zero on sets with interior. For such networks, it is convenient to look at the effective interactions between units rather than the (fixed) structural connectivity to understand the network dynamics. For example, oscillators may effectively decouple in particular phase configurations. Along trajectories, the effective interactions are not necessarily static, but the effective coupling may evolve in time. Here, we formalize the concepts of dead zones and effective interactions. We elucidate how the coupling function shapes the possible effective interaction schemes and how they evolve in time.

This article is part of the theme issue ‘Coupling functions: dynamical interaction mechanisms in the physical, biological and social sciences’.

## 1. Introduction

Many systems in applied sciences can be seen as systems of coupled units that mutually influence each other, such as interacting neurons of an animal's nervous system. Moreover, the system's functionality often depends on emergent properties of this dynamical system. The dynamical behaviour of a network composed of coupled systems depends on a number of factors. One might wish to separate these factors into three broad categories: firstly, the dynamics of component systems (the *unit dynamics*) in isolation; secondly, the structure of the graph of couplings between the systems (the *network structure* viewed as a directed graph where the nodes are systems and the edges are connections); and, thirdly, the nature of the interactions (we refer, in general, to this as *coupling functions* [1]).

This approach can be limiting in several ways. First, it is well known that coupling isolated systems with simple unit dynamics may result in relatively simple dynamics (such as synchronization); equally, it can result in the emergence of qualitatively different dynamics [2]. While a graph structure—as a graph of connections—is an efficient way to encode linearly weighted coupling between dynamical units, it may not capture higher-order, multi-way coupling. Such interactions are typically not ‘pairwise’ if there are nonlinear interactions between three or more nodes; for example, if the input from unit 2 to unit 1 is modulated by unit 3. This leads to more general *non-pairwise coupling* that has recently been investigated in coupled oscillators (for example [3–5]) and in a broader contexts such as ecological networks [6]. Second, even if one assumes linearly weighted pairwise couplings, the coupling function itself is often assumed to be fairly simple in some sense: in the case of weakly coupled oscillatory units, the interaction is typically never assumed to vanish on any interval of phase differences.

Here, we analyse networks with more complex coupling functions that allow dynamical units to (effectively) dynamically decouple and recouple as the system evolves. More specifically, the dynamical systems we investigate have state-dependent interactions that arise through *dead zones* in the coupling function; this concept has been previously identified (for example [7]). Intuitively speaking, in the dead zones of a coupling function there is no interaction. We call the complements *live zones* and formalize these concepts below. Although a wide range of adaptive and time-dependent networks have been studied in the past, this sort of coupling between network structure and dynamics has been overlooked in many contexts, probably because the coupling functions considered may be thought to be pathological (the functions have non-trivial variation at some regions and trivial variation at others and so cannot be analytic). State-dependent dynamics induced by coupling with dead zones relate dynamical models in systems biology, through piecewise linear dynamics characterized by thresholds (for example [8–13]) or continuous modelling (e.g. [14,15]). However, the investigation in these specific settings have focused almost exclusively on the asymptotic behaviour (whether synchronized, periodic [16–19] or chaotic [20,21]) of the underlying high-dimensional coupled system rather than on the dynamics of the effective interactions between individual subsystems of a network. This is also the case in [22,23], where the authors have related the existence of circuits in the graph of interactions to the existence of multistable or stable limit cycles in phase space.

In this paper, we elucidate the interplay between dynamics and *effective* interactions in networks of coupled phase oscillators. We first define the notion of a dead zone for such systems, which leads to the definition of an effective coupling graph at a particular state. The network dynamics are determined by the effective interactions: the dynamics at a particular point are determined by the (state-dependent) effective interactions which may change over time. Hence, the dynamics are determined by both the structural properties (what effective interactions are possible) and the dynamical properties (whether and how do the effective interactions change over time) of the system. Our contribution is threefold. First, we consider the structural question what effective interaction graphs are possible for a given network structure by careful design of coupling functions and examination of the dead zones. Second, we give a result on how the effective interactions do (or do not) change as time evolves. Third, we give instructive examples how dead zones shape the network dynamics; in particular, for a fully connected network and a coupling function for which the dynamics are fully understood, a single dead zone can induce non-trivial periodic dynamics.

While we restrict ourselves to dead zones in coupled phase oscillators, we note that these concepts are likely to be applicable to more general network dynamical systems in a wider range of contexts. Indeed, even in networks that appear structurally simple (for example, networks that are all-to-all coupled from a structural point of view) the existence of dead zones can induce new dynamics. A decomposition of phase space into regions of identical effective interactions yields a natural coarse-graining of the system: it can be understood in terms of the transitions between effective interactions similar to the state transition diagrams in [9]. Such a dynamical decomposition can provide a framework for network dynamical systems with coupling that has ‘approximate’ dead zones—regions where the coupling is small but non-zero. Hence, we anticipate that notable examples and applications may arise in systems biology as discussed above, where many studies use this type of active/inactive interactions, neuroscience, where for example state- and time-dependent interactions may arise, for example, through mechanisms such as spike-time-dependent plasticity [24] or refractory periods, or continuous opinion models [25,26] where agents only interact if their opinion is sufficiently close.

### (a) Dead zones for phase oscillators

The particular class of system that we study in this paper has particularly simple unit dynamics (coupled phase oscillators) and pairwise coupling so that a network of interactions and pairwise coupling functions are appropriate. These models arise naturally in a range of applications where there are coupled limit cycle oscillators and the coupling is weak compared to the limit cycle stability [27,28]. More precisely, we assume that the phase $\theta_{k}\in T:=R/(2\pi Z)$ of oscillator k∈{1,…,N} evolves according to

1.1 $${\dot{\theta }}_{k}=\omega +\sum_{j=1}^{N}A_{jk}g(\theta_{j}-\theta_{k}),$$

where *ω* is the fixed intrinsic frequency of all oscillators, $A_{jk}\in \left\{0,1\right\}$ encode the coupling topology between oscillators (we assume no self-coupling, *A*_*kk*_ = 0), and the (non-constant) *coupling function* g: T→R determines how the oscillators influence each other. A graph ***A*** is associated with the adjacency matrix (*A*_*jk*_)—the *structural coupling graph* that encodes whether oscillator *k* can receive input from oscillator *j*. We constrain ourselves here to phase oscillator networks where the oscillators have the same intrinsic frequency and the coupling function g is the same between all pairs of oscillators.

The coupling topology relates to dynamical properties of network dynamical systems such as (1.1). For commonly studied coupling functions, properties of the structural coupling graph ***A*** such as its spectrum [29,30] determine, for example, synchronization properties (complete or partial) of the network (1.1): this is used in the master stability function approach of Pecora & Caroll [31,32] and the work of Wu & Chua [33], who revealed the role played by the spectral gap and the spectral radius of ***A***. Various conditions for complete synchronization of networks with arbitrary graph structures have been found using spectral properties [34–38]. Consequently, this helps understand the effects of structural perturbations on the synchronizability of networks, including (1.1) and networks where the unit dynamics are more complex (see [39–43]).

Note that it is not necessarily sufficient to consider the structural coupling graph ***A*** to determine dynamical properties: this is particularly the case if the coupling function g has *dead zones*, i.e. if it is zero over some interval of phase differences. In the presence of dead zones, we will define an *effective coupling graph* of (1.1) as a subgraph of ***A***, which encodes the effective interactions between oscillators at a particular point in phase space. By definition, the effective coupling graph is state-dependent and may change dynamically with time. As the system evolves, the network may even decouple into several components under the influence of dead zones. These networks with time- and state-dependent links can also be viewed in the framework of ‘asynchronous networks’ [44,45].

Even though we assume that the uncoupled units are very simple and the functional form of interactions is the same, the possible dynamics of (1.1) may be very complex [28]. We, therefore, mostly restrict equation (1.1) to the case where the coupling is all-to-all (and thus fully symmetric), that is *A*_*kj*_ = 1 for all *j*≠*k*, and the phase $\theta_{k}\in T$ evolves according to

1.2 $${\dot{\theta }}_{k}=\omega +\sum_{j=1,j\neq k}^{N}g(\theta_{j}-\theta_{k})$$

for *k* = 1, …, *N*. In spite of the high degree of symmetry of the system, the system shows a very rich variety of behaviour that includes synchronization [46], clustering [47], heteroclinic dynamics [48] and chaos [49]; see also [47,50,51] for a discussion of the dynamics and bifurcations of (1.2) and see [52] for a recent review.

Looking at the structural coupling graph, the network associated with (1.2) is rather simple, since the network is *fully connected*. However, this also means that there is a rich set of 2^*N*(*N*−1)^ subgraphs corresponding to setting (off-diagonal) entries of ***A*** to zero. For a coupling function with dead zones, this means there is a very rich set of effective coupling graphs that can occur.

**Figure 1.**
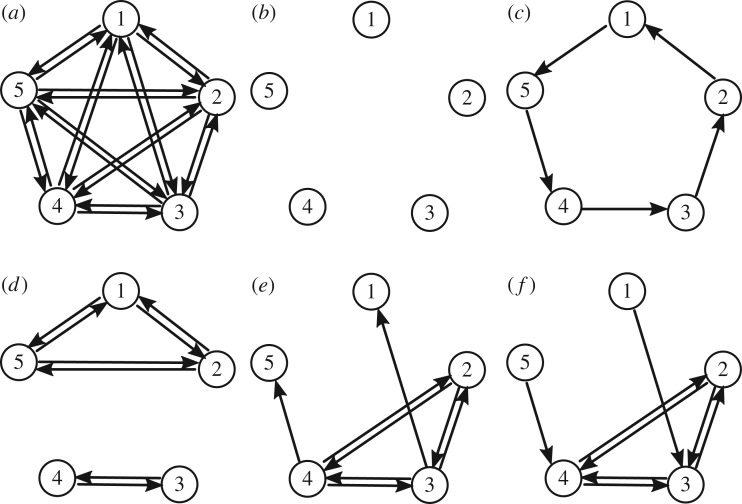
(*a*) Coupling for the graph ***K***_5_ corresponding to the fully connected network (1.2) with *N* = 5. Panels (*b*–*f* ) show five examples of the 2^5×4^ = 1 048 576 possible embedded subgraphs of (*a*), i.e. having the same number of nodes as (*a*): by proposition 3.4, we can show that all of these and more can be realized as effective coupling graphs for a coupling function g with dead zones. Panels (*b*,*d*) show graphs with more than one component: (*b*) is the ‘empty’ graph with no edges, (*c*) is a cycle of length 5 and (*d*,*f* ) have non-trivial structure. A typical trajectory of the system for such a system will visit several different effective coupling graphs under time-evolution. While (*e*,*f* ) shows similar structure proposition 4.3 shows that only (*e*) can be realized in a dynamically stable manner as it contains a spanning diverging tree.

Our goal here is to explore some connections between properties of such dead zones, the effective coupling and the typical dynamics for such networks with these coupling functions for networks of the form (1.1) or (1.2). As an example, figure 1 shows some possible effective couplings that can be achieved by (1.2) with *N* = 5 and choices of coupling function with dead zones. Section 2 presents a setting in which these dead zones can be defined. It also presents conditions in proposition 2.12 for local skew product structure that appears in the dynamics due to the dead zones. We then address the following questions:

Q0 Given any subgraph of the structural coupling graph, is there a coupling function such that this subgraph is *realized* as the effective coupling graph for some point in the phase space?
Q1 What is the relation between the coupling function, the set of possible subgraphs that can be realized, and the points where these realizations happen?
Q2 How do the dynamics and effective couplings influence each other?

Section 3 discusses some results related to these questions concerning effective coupling graphs: proposition 3.4 answers Q0 positively in that it shows that any directed graph can be realized as an effective coupling for a suitable coupling function. A refinement of this proposition is given by proposition 3.8 by showing it is possible to do it at a given length of live zone (small enough with respect to the number of nodes). We also consider specific subcases of Q1 by showing how symmetries of the points in the torus determine partially the effective coupling graphs. Section 4 moves on to Q2 and consider the interaction between effective coupling and dynamics: proposition 4.3 (and corollary 4.4) show that any graph that admits a spanning diverging tree can not only be realized at some point in phase space but also this can be made dynamically stable. More generally, it seems that the interaction of dynamics and dead zones may be quite complex and so we explore some examples. Section 5 looks in detail at the realized effective coupling graphs for *N* = 2 and *N* = 3 with one dead zone. Finally, §6 explores uses, generalizations and applications of these ideas.

### (b) Graph theoretic preliminaries

We briefly introduce some notions (and notation) that are used in this paper (see, e.g., [53] for more background on graph theory). Recall that a *directed graph* ***G*** is a pair ***G*** = (*V*, *E*) with a finite set of *vertices* *V* and directed *edges* *E*⊂*V*^2^ between vertices. Depending on the context, we write *V* (***G***), *E*(***G***) to denote the vertices and edges of ***G***. A pair (*j*, *k*)∈*E* is an *edge from vertex* *j* *to vertex* *k*. Since the graphs we consider here relate to network dynamics, we use terms *vertex/node* and *edge/link* interchangeably. We will assume that the graphs do not contain self-loops, i.e. (*k*, *k*)∉*E* for any *k*. A vertex *k*∈*V* is said to have an incoming edge if there exists another vertex *j* such that (*j*, *k*)∈*E*. Any graph ***G*** can be identified with an *adjacency matrix* *A*^***G***^ with coefficients *A*^***G***^_*kj*_ = 1 if (*k*, *j*)∈*E*(***G***) and *A*^***G***^_*kj*_ = 0 otherwise. We say the graph ***G*** is *undirected* if *A*^***G***^ is symmetric, i.e. *A*^***G***^ is equal to its transpose, or equivalently, (*j*, *k*)∈*E* if and only if (*k*, *j*)∈*E*. This means that an undirected graph has an even number of directed edges. Finally, a graph ***H*** = (*V* ′, *E*′) is a *subgraph of* ***G***, and we write ***H***⊂***G***, if *V* ′⊂*V* and *E*′⊂*E*. In this paper, it will be convenient to consider a subgraph (*V* ′, *E*′)⊂***G*** as an *embedded subgraph* (*V*, *E*′) by including all vertices *V* of ***G***. As an example, figure 1*a* illustrates the fully connected graph on five vertices and figure 1*b*–*f* some embedded subgraphs.

Write $V_{N}=\left\{1,\ldots ,N\right\}$. If ***G*** = (*V*_*N*_, *E*) and ***H***⊂***G*** is an embedded subgraph, then the associated adjacency matrices *A*^***G***^, *A*^***H***^ are *N* × *N* matrices. The *fully connected graph* ***K***_*N*_ is the graph on *V*_*N*_ with (*j*, *k*)∈*E*(***K***_*N*_) for all *j*≠*k*; it has *N*(*N* − 1) edges (figure 1*a*). Similarly, let **∅**_*N*_ = (*V*_*N*_, ∅) denote the *empty graph* with no edges; cf. figure 1*b*. Note that ***K***_*N*_ and **∅**_*N*_ are undirected (figure 1). For (*p*_1_, *p*_2_, …, *p*_*r*_)∈*V*^*r*^_*N*_, let ***P***_*p*_1_,…,*p*_*r*__ denote the *directed path* with vertices *p*_1_, …, *p*_*r*_, i.e. the subgraph of ***K***_*N*_ with edge set

$$E(P_{p_{1},\ldots ,p_{r}})=\left\{(p_{q},p_{q+1})\;|\;q=1,\ldots ,r-1\right\}$$

and similarly, let ***C***_*p*_1_,…,*p*_*r*__ be the *directed cycle* with vertices *p*_1_, …, *p*_*r*_ and edges $E(C_{p_{1},\ldots ,p_{r}})=E(P_{p_{1},\ldots ,p_{r}})\cup \left\{(p_{r},p_{1})\right\}$; cf. figure 1*c*. The *undirected path* ${\bar{P}}_{p_{1},\ldots ,p_{r}}$ and *undirected cycle* ${\bar{C}}_{p_{1},\ldots ,p_{r}}$ are obtained by adding the reverse edges to ***P***_*p*_1_,…,*p*_*r*__, ***C***_*p*_1_,…,*p*_*r*__, respectively. Finally, let ***K***_*p*_1_,…,*p*_*r*__ be the *fully connected subgraph* on the set of nodes {*p*_1_, …, *p*_*r*_}. When convenient, we will identify the graphs ***P***_*p*_1_,…,*p*_*r*__, ***C***_*p*_1_,…,*p*_*r*__ and ***K***_*p*_1_,…,*p*_*r*__ (and their undirected versions) with their corresponding embedded subgraphs with vertices *V*_*N*_.

A directed graph ***G*** = (*V*, *E*) is *strongly connected* if given any two vertices in *V* there exists a directed path of edges in *E* between these two nodes. A directed graph ***G*** is said to be *weakly connected* if it is not strongly connected and its underlying undirected graph, obtained by ignoring the orientations of the edges, is strongly connected. A *spanning diverging tree of ***G*** = (*V*, *E*)* is a weakly connected subgraph of ***G*** such that one node (the root node) has no incoming edges and all other nodes have one incoming edge (for instance, the graph in figure 1*e* contains such a tree). Lastly, for any graph ***G*** = (*V*, *E*), an *independent set* *S* is a set of nodes included in *V* , for which any two nodes of *S* are never connected by an edge in *E*. We say that ***G*** is a *k*-partite graph if *V* admits a partition in *k* distinct independent sets.

### (c) Symmetries, dynamics and graphs

Let ***G*** be a graph with *V* (***G***) = *V*_*N*_ and let $S_{N}$ be the symmetric group of all permutations of $V_{N}=\left\{1,\ldots ,N\right\}$. The *automorphisms of* ***G***, denoted by

$$\Gamma (G)=\gamma \in S_{N}\;|\;A{\text{​}}_{\gamma (k)\gamma (j)}=A_{jk}\;\text{for all }j,k\in V_{N}\},$$

form a subgroup of $S_{N}$ under composition. Define the set of embedded subgraphs

$$H(G)=\left\{H=(V_{N},E^{\prime })\;|\;H\subset G\right\}$$

and write $H_{N}=H(K_{N})$. Note that the group *Γ*(***G***) naturally acts on H(G): For H∈H(G) and *γ*∈*Γ*(***G***) the image *γ****H*** is the graph with vertices *V*_*N*_ and edges

E(γH)={(γ(j),γ(k)) | (j,k)∈E(H)}

for *γ*∈*Γ*(***G***). For this action, the *isotropy group of the graph* ***H***⊂***G*** is

$$\Sigma_{H}=\left\{\gamma \in \Gamma (G)\;|\;\gamma H=H\right\}.$$

Note that the isotropy group does not uniquely identify the subgraph, for example one can reverse the edges and get the same isotropy; however, it is a useful characterization of the graph.

The group $S_{N}$ acts on $T^{N}$ by permuting components. Let $G\in H_{N}$. For $\Sigma \subset \Gamma (G)\subset S_{N}$ we define the *fixed point space* $Fix(\Sigma )=\{\;\theta \in T^{N}\;|\;\gamma (\theta )=\theta \text{ for all }\gamma \in \Sigma \}$. For a given $\theta \in T^{N}$, the *isotropy subgroup* of *θ* is $\Sigma_{\theta }=\left\{\gamma \in \Gamma (G)\;|\;\gamma (\theta )=\theta \right\}$. The symmetries have a number of dynamical consequences for the oscillator network (1.1) for ***G*** above being the structural coupling graph ***A***: note that (1.1) is equivariant with respect to the action of Γ(A)×T where $\Gamma (A)\subset S_{N}$ acts via permutation of the oscillators and ϕ∈T acts via phase shifts

1.3 $$(\theta_{1},\ldots \theta_{N})\mapsto (\theta_{1}+\phi ,\ldots \theta_{N}+\phi ).$$

The fixed point space of any isotropy subgroup of Γ(A)×T is dynamically invariant [50]. It is often useful to consider behaviour of (1.1) in terms of the group orbits of T. Equivalently, the quotient by T corresponds to considering the dynamics in phase difference coordinates, and relative equilibria (equilibria for the quotient system) typically correspond to periodic orbits for the original system.

For the structural coupling graph ***A*** = ***K***_*N*_, we obtain the all-to-all coupled oscillator network (1.2), which is $\Gamma (K_{N})\times T=S_{N}\times T$ equivariant. In this case, the dynamics on the full phase space $T^{N}$ are completely determined by the dynamics on the *canonical invariant region (CIR)* [50,51]

1.4 $$C=\left\{\theta =(\theta_{1},\ldots ,\theta_{N})\;|\;\theta_{1}<\theta_{2}<\cdots <\theta_{N}<2\pi \right\}.$$

The full synchrony and splay phase configurations

$$\Theta^{\mathrm{sync}}=(\phi ,\ldots ,\phi ),\;\Theta^{\mathrm{splay}}=\left(\phi ,\phi +\frac{2\pi }{N},\ldots ,\phi +\frac{(N-1)2\pi }{N}\right)\in C$$

are relative equilibria of the dynamics. There is a residual action of $Z_{N}:=Z/NZ$ on the CIR and *Θ*^splay^ is the fixed point of this action [50].

## 2. From dead zones to effective coupling graphs

In this section, we define dead zones for a coupling function and introduce the resulting effective coupling graph and its properties. We will restrict to a suitable class of coupling functions that have dead zones but are otherwise smooth and in general position; clearly, this could be easily generalized, for example to coupling functions with only finite differentiability.

**Definition 2.1.**

Suppose that g: T→R is a smooth 2*π*-periodic function.

— A coupling function g is *locally constant at $\theta^{0}\in T$ with value c∈R* if there is an open set *U* with $\theta_{0}\in U\subset T$ such that g(*U*)≡*c*. Define LC(g) to be the set of locally constant points of g.
— A coupling function g is *locally null at $\theta^{0}\in T$* if it is locally constant with *c* = 0. Let DZ(g)⊂LC(g) denote the set of locally null points of g.
— A coupling function g has *simple dead zones* if DZ(g) has finitely many connected components and LC(g) = DZ(g), i.e. if there is a finite set of locally constant regions, and all are locally null.

**Definition 2.2.**

Let g be a coupling function with simple dead zones. Any connected component of DZ(g) is a *dead zone* of g. Connected components of the complements LZ(g)=T∖DZ(g) are *interaction* or *live zones*.

Here, we will only consider the case of *simple dead zones*: in the rest of the paper, we will implicitly consider only *coupling functions with simple dead zones*. The class of coupling function with simple dead zones excludes (smooth approximation of) piecewise constant coupling functions. These may have non-trivial dynamics that are solely given by the different frequencies in the region where the coupling function is locally constant. Such non-trivial dynamics are clearly of interest in some applications, but is beyond the scope of this paper.

**Definition 2.3.**

We say that g is *dead zone symmetric* if −DZ(g) = DZ(g) modulo 2*π*, i.e. if whenever ϕ∈T is in a dead zone, then −*ϕ* also belongs to a dead zone.

As an illustration, figure 7 in §5 provides examples of coupling functions (which are dead zone symmetric or not) with one dead zone.

### (a) Effective coupling graphs and their symmetries

Suppose that g is a coupling function for (1.1) with structural coupling graph ***A*** given by the adjacency matrix (*A*_*jk*_) and let $\theta \in T^{N}$. We say a node *k* is g-*effectively influenced by node j at* *θ* for (1.1) if *A*_*jk*_ = 1 and *θ*_*j*_ − *θ*_*k*_∉DZ(g).

**Definition 2.4.**

The *effective coupling graph* $G_{g,A}(\theta )$ of (1.1) with coupling function g at $\theta \in T^{N}$ is the graph on *N* vertices with edges

$$E(G_{g,A}(\theta ))=\left\{(j,k)\;\left|\;A_{jk}\neq 0\;\text{and}\;\theta_{j}-\theta_{k}\notin \text{DZ}(g\right)\right\}.$$

Conversely, an edge $\left(j,k)\notin E(G_{g,A}(\theta )\right)$ if *A*_*jk*_ = 0 (the edge is not contained in ***A***) or *θ*_*j*_ − *θ*_*k*_∈DZ(g) (the phase difference is in a dead zone).

Clearly $G_{g,A}(\theta )\subset A\subset K_{N}$, and this will be a proper subgraph (that is, it differs from ***A*** by at least one edge) for some $\theta \in T^{N}$ if g has at least one dead zone. For the system (1.1) with coupling function g and given ***H***⊂***K***_*N*_, define

2.1 $$\Theta_{g,A}(H)=\left\{\theta \in T^{N}\;|\;G_{g,A}(\theta )=H\right\}.$$

**Definition 2.5.**

If *Θ*_g, ***A***_(***H***) is not empty, then ***H*** is *realized as an effective coupling graph for (1.1) with coupling function g*. Moreover, a graph ***H*** *can be realized as an effective coupling graph for (1.1)* if there exists a coupling function g for which *Θ*_g, ***A***_(***H***) is not empty.

For the special case ***A*** = ***K***_*N*_, that is the oscillator network (1.2), we simply write $G_{g}(\theta )$ for the effective coupling graph with edges

$$E(G_{g}(\theta ))=\left\{(j,k)\;\left|\;\theta_{j}-\theta_{k}\notin \text{DZ}(g\right)\right\}.$$

Similarly, we write

2.2 $$\Theta_{g}(H)=\left\{\theta \in T^{N}\;|\;G_{g}(\theta )=H\right\}$$

for the regions of phase space with a particular effective coupling graph. Note that the sets *Θ*_g_(***H***), $H\in H_{N}$, partition the CIR C.

For particular structural coupling graphs ***A*** of (1.1), there is a large number of symmetries, i.e. the automorphism group *Γ*(***A***) may be large [31]; it is maximal for ***A*** = ***K***_*N*_. At the same time, *Γ*(***A***) acts on the underlying phase space. We now show how the symmetry of a point $\theta \in T^{N}$ relates to the symmetries of the effective coupling graph at *θ*.

**Lemma 2.6.**

*Consider the system* (*1.1*) *with structural coupling graph* ***A*** *and any coupling function* g. *For any* $\theta \in T^{N}$, *we have* $G_{g,A}(\gamma \theta )=\gamma G_{g,A}(\theta )$ *for all* *γ*∈*Γ*(***A***).

**Proof.**

Note that [*γθ*]_*k*_ = *θ*_*γ*(*k*)_, where [  ·  ]_*k*_ refers to the *k*th component, and so [*γθ*]_*j*_ − [*γθ*]_*k*_∈DZ(g) if and only if *θ*_*γ*(*j*)_ − *θ*_*γ*(*k*)_∈DZ(g). These are the edges of $\gamma G_{g,A}(\theta )$. ▪

**Corollary 2.7.**

*Consider the system* (*1.1*) *with structural coupling graph* ***A*** *and any coupling function* g. *For any* $\theta \in T^{N}$, *we have*

$$\Sigma_{\theta }\subset \Sigma_{G_{g,A}(\theta )}\subset \Gamma (A).$$

**Proof.**

To see this, note that if *γ*∈*Σ*_*θ*_ then *γθ* = *θ* and so $G_{g,A}(\theta )=G_{g,A}(\gamma \theta )=\gamma G_{g,A}(\theta )$ which implies that $\gamma \in \Sigma_{G_{g,A}(\theta )}$. ▪

Note that the reverse containment of corollary 2.7 does not necessarily hold, for example if there are no dead zones (i.e. if DZ(g) is empty) then clearly *Σ*_*θ*_ = *Γ*(***A***) for all *θ*.

**Remark 2.8.**

Note that while C is a fundamental region for the dynamics of the all-to-all coupled network (1.2), the effective coupling graphs can differ between symmetric copies of C.

### (b) Local (skew-)product structure and asynchronous networks

In this section, we show that the effective coupling graph at a point captures essential dynamical information. In particular, we have that, locally around a generic point, the vector field factorizes into factors that correspond to (weakly) connected components of the effective coupling graph.

For $v=\left\{v_{1}<v_{2}<\cdots <v_{r}\right\}\subset V_{N}$ let $\pi_{v}:T^{N}\to T^{r}$ denote the projection of $T^{N}$ onto the coordinates in *v*. We write $T^{v}:=\pi_{v}(T^{N})$ and *θ*_*v*_ = (*θ*_*v*_1__, …, *θ*_*v*_*r*__) are the coordinates in $T^{v}$. Suppose that *v*^1^, *v*^2^⊂*V*_*N*_ partition *V*_*N*_, that is, *v*^1^∩*v*^2^ = ∅, *v*^1^∪*v*^2^ = *V*_*N*_. Write *r*_*k*_ = |*v*^*k*^| for the length of the vector *v*^*k*^ and we identify $T^{N}≅T^{r_{1}}\times T^{r_{2}}$ with elements *θ* = (*θ*_*v*^1^_, *θ*_*v*^2^_) through the natural isomorphism that reorders coordinates appropriately.

**Definition 2.9.**

Consider a general ODE on the *N*-torus,

2.3 $$\dot{\theta }=F(\theta ),$$

with $\theta \in T^{N}$, $F:\;T^{N}\to T^{N}$ some smooth function, and *v* = (*v*^1^, *v*^2^) a partition of *V*_*N*_.

(a) The system has a *local skew-product structure* *v*^1^ → *v*^2^ at $\theta \in T^{N}$ if there is an open neighbourhood *U* of *θ* and functions *F*_(1)_, *F*_(2)_ such that *F*(*θ*) = (*F*_(1)_(*θ*_*v*^1^_), *F*_(2)_(*θ*_*v*^1^_, *θ*_*v*^2^_)) for all *θ*∈*U*.
(b) The system has a *local product structure with respect to* *v* at $\theta \in T^{N}$ if there is an open neighbourhood *U* of *θ* and functions *F*_(1)_, *F*_(2)_ such that *F*(*θ*) = (*F*_(1)_(*θ*_*v*^1^_), *F*_(2)_(*θ*_*v*^2^_)) for all *θ*∈*U*.

The second statement is equivalent to *F* having a local skew product structure *v*^1^ → *v*^2^ and *v*^2^ → *v*^1^ at *θ*. For $A\subset T^{N}$ let int(*A*) denote the interior of *A*.

**Lemma 2.10.**

*Consider the dynamics* (*1.1*) *with a coupling function* g. *Generically*, *θ*∈ int(*Θ*_g, ***A***_(***H***)) *for some* ***H***.

**Proof.**

We have that

$$T^{N}\setminus \underset{H\subset G}{⋃}\text{int}(\Theta_{g,A}(H))=\left\{\theta =(\theta_{1},\ldots ,\theta_{N})\in T^{N}\;|\;\exists k\neq j:\theta_{k}-\theta_{j}\in \partial \text{DZ}(g)\right\}$$

is a union of finitely many algebraic sets. ▪

Note that this does not imply that all *Θ*_g, ***A***_(***H***) have non-empty interior. Consider for example the fully connected network (1.2) with ***A*** = ***K***_*N*_ and a coupling function g such that 0∈∂DZ(g). Then *Θ*_g_(***K***_*N*_) has an isolated point.

**Lemma 2.11.**

*Consider the dynamics of* (*1.1*) *written in the form* (*2.3*) *for a coupling function* g. *Suppose that* *θ*∈int(*Θ*_g, ***A***_(***H***)). *Then* (*j*, *k*)∉*E*(***H***) *if and only if there exists a neighbourhood* $U\subset T^{N}$ *of* *θ* *such that* (∂_*θ*_*j*__*F*_*k*_)(*θ*) = 0 *for all* *θ*∈*U*.

**Proof.**

Write *θ* = (*θ*_1_, …, *θ*_*N*_)∈int(*Θ*_g, ***A***_(***H***)). First, suppose that (*j*, *k*)∉*E*(***H***). Since *θ*∈int(*Θ*_g, ***A***_(***H***)) there exists a neighbourhood *U*⊂int(*Θ*_g, ***A***_(***H***)) of *θ*. Now (∂_*θ*_*j*__*F*_*k*_)(*θ*) = *A*_*kj*_*g*′(*θ*_*j*_ − *θ*_*k*_) = 0 for all *θ* = (*θ*_1_, …, *θ*_*N*_)∈*U* since either *A*_*kj*_ = 0 or *θ*_*j*_ − *θ*_*k*_∈DZ(g). Conversely, suppose that there exists an *U* such that ∂_*θ*_*j*__*F*_*k*_ = 0 on *U*. But 0 = ∂_*θ*_*j*__*F*_*k*_ = *A*_*kj*_*g*′(*θ*_*j*_ − *θ*_*k*_) on *U* which implies *A*_*kj*_ = 0 or *g*′(*θ*_*j*_ − *θ*_*k*_) = 0 (on *U*). In either case, we have (*j*, *k*)∉*E*(***H***) by definition. ▪

Let ***G*** = (*V*, *E*) be a graph. A partition {*v*^1^, *v*^2^} of *V* is a *graph cut*. Write $E_{v^{p}\to v^{q}}(G)=\left\{(j,k)\in E(G)\;|\;j\in v^{p},k\in v^{q}\right\}$ for the edges from vertices in *v*^*p*^ to vertices in *v*^*q*^. The *cut-set* of {*v*^1^, *v*^2^} is *E*_*v*^1^ → *v*^2^_(***G***)∪*E*_*v*^2^ → *v*^1^_(***G***). The graph cut is *directed *v*^*p*^ → *v*^*q*^* if *E*_*v*^*p*^ → *v*^*q*^_(***G***)≠∅, *E*_*v*^*p*^ → *v*^*q*^_(***G***) = ∅. The partition is *disconnected* if *E*_*v*^*p*^ → *v*^*q*^_(***G***) = *E*_*v*^*p*^ → *v*^*q*^_(***G***) = ∅. The following result relates properties of the effective coupling graph at a given point *θ* with the local properties of the dynamical system (1.1).

**Proposition 2.12.**

*Consider a generic point* $\theta \in T^{N}$.

(i) *There is a directed graph cut* *v*^1^ → *v*^2^ *for the effective coupling graph* $G_{g,A}(\theta )$ *iff the system* (1.1) *has a local skew-product structure* *v*^1^ → *v*^2^ *at* *θ*.
(ii) The partition {*v*^1^, *v*^2^} of the effective coupling graph $G_{g,A}(\theta )$ is disconnected iff the system (1.1) has a local product structure *v*^1^ → *v*^2^ at *θ*.

**Proof.**

Write $H=G_{g,A}(\theta )$. By lemma 2.10, we may assume *θ*∈int(*Θ*_g, ***A***_(***H***)). Suppose that {*v*^1^, *v*^2^} is a partition. The assertion now follows from applying lemma 2.11 for any edge in *E*_*v*^1^ → *v*^2^_(***H***) and *E*_*v*^2^ → *v*^1^_(***H***), respectively. ▪

Recall that two nodes *v*, *w* in a directed graph are weakly connected if there is a path of edges (irrespective of their direction) between them. A weakly connected component is a maximal weakly connected subgraph. The following is a direct consequence of proposition 2.12.

**Corollary 2.13.**

*Consider a generic point* $\theta \in T^{N}$ *and let* $H=G_{g}(\theta )$. *If* *v*^1^, …, *v*^ℓ^ *is the partition of the vertices corresponding to the weakly connected components of* ***H*** *then there is a neighbourhood* *U* *of* *θ* *such that* (*1.1*) *can be written in the form* (*2.3*) *with*

$$F(\theta )=(F_{v^{1}}(\theta_{v^{1}}),\ldots ,F_{v^{\ell }}(\theta_{v^{\ell }}))$$

*on* $U\subset T^{v^{1}}\times \cdots \times T^{v^{\ell }}≅T^{N}$.

**Remark 2.14.**

A network of coupled oscillators naturally defines a non-trivial asynchronous network as defined in [44]. Events occur when the effective coupling graph changes along a trajectory, which defines the ‘event map’ $E(\theta )=G_{g,A}(\theta )$. Now the coupled oscillator network (1.1) written as (2.3) is determined by the state-dependent ‘network vector field’ $F(\theta )=F^{E(\theta )}(\theta )$. Moreover, the network structure is ‘additive’ in the sense that the dynamics of each oscillator is determined by a sum of the contributions from other oscillators and the condition whether $\left(j,k)\in G_{g,A}(\theta \right)$ only depends on (*θ*_*k*_, *θ*_*j*_). In the language of [44,45], this means that the asynchronous network is ‘functionally decomposable’ and ‘structurally decomposable’. Finally, note that proposition 2.12 implies that, generically, we have a local product structure, a condition for the spatio-temporal decomposition of the factorization of dynamics theorem [45].

## 3. Realizing effective coupling graphs

In this section, we aim to relate dead zones and effective coupling graphs, noting that the effective coupling graph $G_{g}(\theta )$ depends both on coupling function g and choice of *θ*. Recall that Q0 and Q1 in §1a concern the set of effective coupling graphs that can be realized and the goal of this section is to tackle such *structural* problems. Recall that if *Θ*_g, ***A***_(***H***)≠∅ then we say that the effective coupling graph ***H*** is *realized* for g. Proposition 3.4 answers Q0 in the positive: for typical choice of *θ* (with trivial isotropy), all effective coupling graphs can be realized. We consider two special cases of Q1:

Q1a Given a point $\theta \in T^{N}$, a structural coupling graph ***A*** and a graph H∈H(A), is there a coupling function g for (1.1) (resp. (1.2)) such that $G_{g,A}(\theta )=H$? (resp. $G_{g}(\theta )=H$)?
Q1b Is there a coupling function that realizes all possible effective coupling graphs? Specifically, for (1.2), is there a coupling function g such that^1^ $G_{g}(T^{N})=H_{N}$?

Proposition 3.1 gives some constraints on answers of Q1a. We do not have a complete answer to Q1a, while corollary 3.5 answers Q1b positively. We also consider what possible effective coupling graphs will be realized for a coupling function g: this is important if we wish to understand the dynamics of (1.2) with a fixed coupling function. To some extent this is simply a computational question: for any *θ* = (*θ*_1_, …, *θ*_*N*_) one has to determine which phase differences *θ*_*j*_ − *θ*_*k*_ lie in a dead zone. We give some general results in this direction in §3b. We believe that typical coupling functions will not be able to realize more than a small subset of effective coupling graphs. Henceforth, we mainly restrict to discussion of the fully connected network (1.2) although several of the results easily generalize to (1.1).

### (a) Restrictions on the effective coupling graph imposed by *θ*

Here, we tackle the questions Q1a, Q1b above by putting the emphasis on the point *θ*. Specifically, given $\theta \in T^{N}$, what do the properties of *θ* impose on the effective coupling graphs of (1.2)? The isotropy of $\theta \in T^{N}$ has some important consequences on the possible effective coupling graphs realized at *θ*:

**Proposition 3.1.**

*Consider the all-to-all coupled oscillator network* (*1.2*) *with coupling function* g. *Let* $\theta \in C\subset T^{N}$ *be fixed*.

(i) *If* *θ* *has isotropy* *Σ*_*θ*_ *then* $G_{g}(\theta )$ *must have at least the same isotropy.*
(ii) For full synchrony *Θ*^sync^ = (*a*, …, *a*) we have $G_{g}(\Theta^{\mathrm{sync}})\in \left\{\emptyset_{N},K_{N}\right\}$.
(iii) Suppose there exists 0 < *a* < 2*π*/*N* such that *θ*_*k*+1_ − *θ*_*k*_ = *a* for any k∈{1,…,N−1}. Then one of the following cases occurs:
  (1) The directed path ***P***_*N*,*N*−1,…,1_ is a subgraph of $G_{g}(\theta )$ but ***P***_1,2,…,*N*_ is not.
  (2) The directed path ***P***_1,2,…,*N*_ is a subgraph of $G_{g}(\theta )$ but ***P***_*N*,*N*−1,…,1_ is not.
  (3) The undirected path ${\bar{P}}_{1,2,\ldots ,N}$ is a subgraph of $G_{g}(\theta )$.
  (4) $G_{g}(\theta )$ is a *n*-partite graph (with *n* = [*N*/2] if *N* is even or *n* = [*N*/2] + 1 if not).

**Proof.**

**Figure 2.**
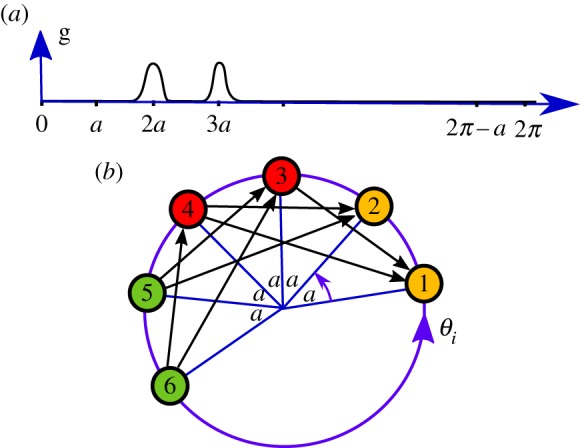
Illustration of case (4) in proposition 3.1(iii) for *N* = 6 oscillators: the coupling function g shown in (*a*) has two live zones centred at 2*a* and 3*a*, the remainder consists of two dead zones. The diagram in (*b*) shows the phases *θ*_*k*_ at one instant in time such that *θ*_*j*_ − *θ*_*i*_ = *a*(*j* − *i*) for all *j* > *i*. The effective coupling graph for the coupling function in (*a*) is indicated by black arrows between the phases corresponding to individual nodes. This coupling graph is tripartite as indicated by the node colouring. (Online version in colour.)

(i) Is an application of corollary 2.7, notably *θ* with non-trivial isotropy will limit the possible networks to those that have at least the same isotropy.

(ii) The claim follows from (i): the effective coupling graph must have full symmetry and hence be either ***K***_*N*_ or **∅**_*N*_.

(iii) We have one of the following cases as (we illustrate case (4) in figure 2): (1) *a*∈LZ(g), 2*π* − *a*∈DZ(g), (2) *a*∈ DZ(g), 2*π* − *a*∈LZ(g), (3) *a*, 2*π* − *a*∈ LZ(g), (4) *a*, 2*π* − *a*∈DZ(g). Since all the differences *θ*_*i*+1_ − *θ*_*i*_ are equal to *a* (and thus all *θ*_*k*_ − *θ*_*k*+1_ equal to 2*π* − *a*), then in case (1) we have that ***P***_*N*,*N*−1,…,1_ is a subgraph of $G_{g}(\theta )$ but not ***P***_1,2,…,*N*_. Similarly for the cases (2) and (3). In the case (4), the vertices of $G_{g}(\theta )$ can be partitioned into *n* = [*N*/2] independent sets *A*_*n*_ if *N* is even (or into *n* = [*N*/2] + 1 such sets if *N* is odd): namely the successive sets $A_{1}=\left\{1,2\right\}$, $A_{2}=\left\{3,4\right\}$, etc. This means that $G_{g}(\theta )$ is a *n*-partite graph. ▪

While proposition 3.1(iii) limits to the splay configuration *Θ*^splay^ in a special case, the next proposition gives a characterization of effective coupling graphs that are realized for *Θ*^splay^.

**Proposition 3.2.**

*Consider the coupled oscillator network* (*1.2*) *with coupling function* g.

(i) *If* 2*π*/*N*∈LZ(g) *then the directed cycle* ***C***_1,2,…,*N*_ *is a subgraph of* $G_{g}(\Theta^{\mathrm{splay}})$.
(ii) Let 1 < *n* < *N* and suppose that 2*nπ*/*N*∈LZ(g). Take indices modulo *N*.
  (a) If *N* = *nm* then for any r∈{1,…,m} the directed cycle ***C***_*r*,*r*+*n*,…,*r*+(*m*−1)*n*_ is a subgraph of $G_{g}(\Theta^{\mathrm{splay}})$.
  (b) If *n* does not divide *N*, then the directed cycle ***C***_1,1+*n*,1+2*n*,…,1+*N*−*n*_ is a subgraph of $G_{g}(\Theta^{\mathrm{splay}})$.

**Proof.**

(i) As in the proof of proposition 3.1(iii), we consider successive phase differences for *Θ*^splay^ = (*θ*_1_, …, *θ*_*N*_). We have *θ*_*k*+1_ − *θ*_*k*_ = *θ*_1_ − *θ*_*N*_ = 2*π*/*N*. Since 2*π*/*N*∈ LZ(g) by assumption, we have $\left(k,k+1)\in E(G_{g}(\Theta^{\mathrm{splay}})\right)$ which proves that $C_{1,2,\ldots ,N}\subset G_{g}(\Theta^{\mathrm{splay}})$.

(ii) A similar argument proves the second assertion. Let 1 < *n* < *N* and since *θ*_*k*_ − *θ*_*k*+*n*_∈LZ(g) we have $\left(k,k+n)\in E(G_{g}(\Theta^{\mathrm{splay}})\right)$. Now suppose that *N* = *nm* + *q* with 0 ≤ *q* < *m*. If *q* = 0 we have *r* + *pn* = *r* mod  *N* for *p* = *m* < *N* which proves case (a). If *q*≠0 then *r* + *pn* = *r* mod  *N* only if p∈NZ which corresponds to case (b). ▪

Recall that *Θ*^splay^ is the only fixed point of the residual action of $Z_{N}$ on the CIR [50]. The graph $G_{g}(\Theta^{\mathrm{splay}})$ is invariant under the action of the symmetry by proposition 3.1(i). In cases (i) and (iib) of proposition 3.2, $G_{g}(\Theta^{\mathrm{splay}})$ contains one cycle involving all vertices, that is mapped to itself. By contrast, in case (iia) of proposition 3.2, there are *m* disjoint cycles that are permuted by the symmetry. The existence of cycles has also some immediate consequences for the connectedness of $G_{g}(\Theta^{\mathrm{splay}})$.

**Corollary 3.3.**

*Consider system* (*1.2*) *with coupling function* g *and suppose that the number of oscillators* *N* *is prime. Then either* $G_{g}(\Theta^{\mathrm{splay}})=\emptyset_{N}$ *or* $G_{g}(\Theta^{\mathrm{splay}})$ *contains a directed cycle involving all* *N vertices*, *and so* $G_{g}(\Theta^{\mathrm{splay}})$ *is strongly connected.*

**Proof.**

If 2*nπ*/*N*∈DZ(g) then $G_{g}(\Theta^{\mathrm{splay}})=\emptyset_{N}$. Now suppose that there is an 1 ≤ *n* < *N* such that 2*nπ*/*N*∈LZ(g). Since *N* is prime, either proposition 3.2(i) and (iib) applies. In either case, $G_{g}(\Theta^{\mathrm{splay}})$ contains a directed cycle involving all *N* vertices. ▪

The next result gives a positive answer to Q0, providing that we avoid ‘non-generic’ choices of *θ*.

**Proposition 3.4.**

*For a generic choice of* $\theta \in T^{N}$, *and for any subgraph* $H\in H_{N}$, *there exists a coupling function* g *such that* $G_{g}(\theta )=H$.

**Proof.**

Generically all the difference terms *θ*_*j*_ − *θ*_*k*_ (when *j*, *k* are ranging in {1,…,N}) are distinct: therefore, we can specify live zones that contain points *θ*_*j*_ − *θ*_*k*_ if and only if the edge (*j*, *k*) is contained in ***H***. ▪

Notice that the proof gives an upper bound on the number of dead and live zones needed to realize a given ***H*** as an effective coupling graph (by choosing a coupling function and a point *θ*), namely the bound given by the number of edges of ***H***. This bound is far from being optimal, notably for very ‘regular’ graphs: for instance for any $\theta \in T^{N}$, the graph ***K***_*N*_ itself can be realized as a $G_{g}(\theta )$ where g has only one live zone and no dead zone. Corollary 3.5 extends the same method of proof to show that one can, in principle, realize all subgraphs using one and the same g.

**Corollary 3.5.**

*There exists a coupling function* g *such that for any subgraph* $H\in H_{N}$ *there exists* $\theta^{0}=\theta^{0}(H)\in T^{N}$ *such that* $G_{g}(\theta^{0})=H$.

**Proof.**

Enumerate all graphs ***H***_*n*_ in $H_{N}$ and choose a set $\{\theta^{n}=(\theta_{1}^{n},\ldots ,\theta_{N}^{n})\in T^{N}\}$ such that all phase differences *θ*^*n*^_*j*_ − *θ*^*m*^_*k*_ are distinct. Now take a coupling function g such that for any *n* we have *θ*^*n*^_*j*_ − *θ*^*n*^_*k*_∈LZ(g) if and only if (*j*, *k*) is in *E*(***H***_*n*_). ▪

A similar proviso holds here: such a constructed g will typically have a very large number of dead zones.

### (b) Coupling functions for an interaction graph

Given a coupling function g, which properties of g imply certain effective coupling graphs realized by g? On the other hand, given θ∈C, a structural coupling graph ***A*** and H∈H(A), how can one construct a coupling function g such that $H=G_{g,A}(\theta )$? Among the different parameters characterizing the coupling function g, the number of dead zones plays a major role in these questions, since it determines the shapes of the resulting effective coupling graphs. We thus make the following definition.

**Definition 3.6.**

Let n∈N. We denote by F(n) the set of coupling functions having *n* dead zones.^2^

**Proposition 3.7.**

*Consider system* (*1.2*) *with coupling function* g.

(i) *The coupling function* g *is dead zone symmetric if and only if all effective coupling graphs for* g *are undirected.*
(ii) Assume that g∈F(1) is dead zone symmetric with LZ(g) = [ − *a*, *a*]. If *a* < 2*π*/*N*, then for any 1 ≤ *k* ≤ *N* and any sequence *k*, …, *k* + *p* in {1,…,N} we have that **∅**_*N*_, ***K***_*N*_ and the embeddings of ${\bar{P}}_{k,\ldots ,k+p}$ and ***K***_*k*,…,*k*+*p*_ can be realized as effective coupling graphs for g. If *a* = 2*π*/*N*, then $K_{N},{\bar{P}}_{1,\ldots ,N},{\bar{C}}_{1,\ldots ,N},$ and the embeddings of graphs ${\bar{P}}_{k,\ldots ,k+p}$ and ***K***_*k*,…,*k*+*p*_ can be realized as effective coupling graphs for g.
(iii) Assume that g∈F(1) is dead zone symmetric with LZ(g) = [*π* − *a*, *π* + *a*] and *a* ≤ 2*π*/*N*. Then **∅**_*N*_ and ***K***_*N*_ can be realized as effective coupling graphs for g.

**Proof.**

(i) This follows directly from the definition of a dead zone symmetric function.

(ii) Suppose that *a* < 2*π*/*N*. Taking θ∈C such that all the successive differences *θ*_*i*+1_ − *θ*_*i*_ are in (*a*, 2*π*/*N*), we have that $G_{g}(\theta )=\emptyset_{N}$: indeed any phase difference *θ*_*j*_ − *θ*_*k*_, with *j* > *k*, will belong to the interval (*a*, 2*π*(*N* − 1)/*N*) and therefore will be in DZ(g). Similarly, taking *θ* such that all the successive differences *θ*_*k*+1_ − *θ*_*k*_ are strictly smaller than *a*/(*N* − 1) we have $G_{g}(\theta )=K_{N}$. Now consider 1 ≤ *k*, *p* ≤ *N* and a sequence *k*, …, *k* + *p* in {1,…,N}. Taking *θ* such that

$$\begin{array}{rl} & \theta_{k+1}-\theta_{k}=\theta_{k+2}-\theta_{k+1}=\cdots =\theta_{k+p}-\theta_{k+p-1}=a/2+\epsilon ,\\ & \theta_{2}-\theta_{1}=\theta_{3}-\theta_{2}=\cdots =\theta_{k}-\theta_{k-1}=a+\epsilon \\ \;\text{and}\; & \theta_{k+p+1}-\theta_{k+p}=\theta_{k+p+2}-\theta_{k+p+1}=\cdots =\theta_{N}-\theta_{N-1}=a+\epsilon , \end{array}$$

where *ϵ* is a sufficiently small positive real number, we have that $G_{g}(\theta )={\bar{P}}_{k,\ldots ,k+p}$. Similarly taking a point θ∈C such that

$$\begin{array}{rl} & \theta_{k+1}-\theta_{k}=\theta_{k+2}-\theta_{k+1}=\cdots =\theta_{k+p}-\theta_{k+p-1}=\epsilon ,\\ & \theta_{2}-\theta_{1}=\theta_{3}-\theta_{2}=\cdots =\theta_{k}-\theta_{k-1}=a+\epsilon \\ \;\text{and}\; & \theta_{k+p+1}-\theta_{k+p}=\theta_{k+p+2}-\theta_{k+p+1}=\cdots =\theta_{N}-\theta_{N-1}=a+\epsilon , \end{array}$$

with *ϵ* small enough, we have in this case that $G_{g}(\theta )=K_{k,\ldots ,k+p}$. If *a* = 2*π*/*N*, then the same reasoning applies.

(iii) This follows along similar lines as (ii). ▪

Observe that, for the particular points *θ* = *Θ*^sync^ and *θ* = *Θ*^splay^ the coupling functions considered in propositions 3.1(i) and 3.2 can be taken in F(0) and F(1), respectively. The proof of proposition 3.4 constructs functions using many dead zones that might be very small. There are various questions one can pose about optimality. For example, given θ∈C, a structural coupling graph ***A*** and a graph H∈H(A), what is the minimum *n* such that there is a g∈F(n) such that $H=G_{g,A}(\theta )$? The proof of proposition 3.4 gives an upper bound to this question in the case of System (1.2), namely *n* ≤ 2^#*E*(***H***)^ (where #*E*(***H***) is the number of edges of ***H***), but does not give any information on the length of the live zones involved, which can possibly be arbitrarily small, and for which one may need to control the size. Proposition 3.8 below gives a lower bound, as a function of the number of nodes *N* (namely *π*/2^*N*−1^), on the length of live zones *δ* > 0 for which it is possible to realize any ***H*** as an effective coupling graph (and this thanks to a coupling function of which live zones have length *δ*).

**Proposition 3.8.**

*Let* 0 < *a* < *π*/2^*N*−1^ *and let* 0 < *δ* < *a*. *Then*, *for any* $\theta \in T^{N}$ *with*

$$\theta_{i+1}-\theta_{i}\geq \theta_{i}-\theta_{1}+a,\;\text{for }i\in \left\{1,\ldots ,N-1\right\},$$

*and*

$$\theta_{N}-\theta_{1}<\pi -a,$$

*and for any* $H\in H_{N}$, *there exists an integer* *n* ≤ #*E*(***H***) *and a coupling function* g∈F(n) *with live zones of length at least* *δ* *such that* $G_{g}(\theta )=H$.

**Proof.**

**Figure 3.**
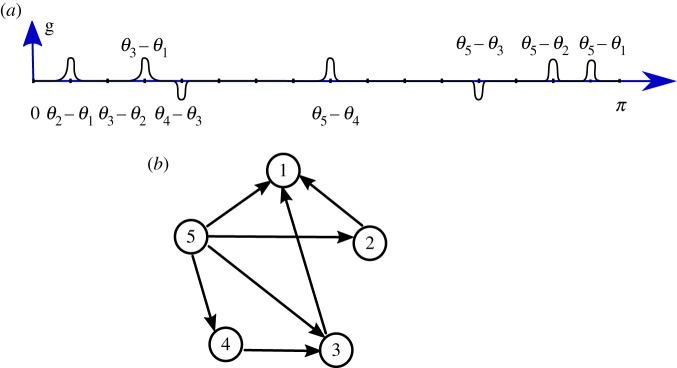
An example of directed graph (*b*) within ***K***_5_ with seven edges realized as an effective coupling graph with a coupling function g in F(7) (*a*) constructed via the method described in the proof of proposition 3.8: in the bottom of the graph of g, we show the successive phase differences *θ*_*i*+1_ − *θ*_*i*_ for which the edge (*i* + 1, *i*) is in *E*(***H***) (the ones for which (*i* + 1, *i*)∉*E*(***H***) are not shown), and at which we impose the existence of a live zone of g. Above the graph of g, we show the values of the phase differences that are determined by the values of the successive phase differences.

Suppose that *θ* satisfies the conditions required and ***H*** is a subgraph of ***G***. The idea is to construct a coupling function for which each live zone is precisely associated with only one edge of ***H*** (figure 3).

To do this, we start with the coupling function identically equal to 0. Now, if (2, 1)∈*E*(***H***) then we put a live zone of length *δ* centred at *θ*_2_ − *θ*_1_. If not, then we set g to be locally null at *θ*_2_ − *θ*_1_. Next, we consider *θ*_3_ − *θ*_2_≥*θ*_2_ − *θ*_1_ + *a*: If (3, 2)∈*E*(***H***) we put a live zone of length *δ* centred at *θ*_3_ − *θ*_2_. This second live zone does not intersect the first one since *θ*_3_ − *θ*_2_ > *θ*_2_ − *θ*_1_ + *δ*. If (3, 2)∉*E*(***H***) we let g be locally null at *θ*_3_ − *θ*_2_. Then we have *θ*_3_ − *θ*_1_≥*θ*_3_ − *θ*_2_ + *a* which satisfies *θ*_3_ − *θ*_1_ > *θ*_3_ − *θ*_2_ + *δ*; we can thus put a live zone of length *δ* centred at *θ*_3_ − *θ*_1_ if (3, 1)∈*E*(***H***).

Repeating the process, we construct g by imposing the existence of a live zone of length *δ* centred at any of the *θ*_*i*_ − *θ*_*k*_ (with 1 ≤ *k* ≤ *i* ≤ *N*) such that (*i*, *k*) is in *E*(***H***). Since all these terms are smaller than *π*, all the opposite values are determined and separated as well by a distance larger than *a*. It is, therefore, possible to add live zones at the *θ*_*k*_ − *θ*_*i*_ (with 1 ≤ *k* ≤ *i* ≤ *N*) for which the edge (*k*, *i*) is in *E*(***H***). ▪

## 4. Dynamics of effective coupling graphs

The previous section considered the structural problem of understanding the effective coupling graph at some point in phase space. Now let *θ*(*t*) = *φ*_*t*_(*θ*^*o*^) be the solution of the phase oscillator network (1.1) with initial condition *θ*^*o*^. Clearly, $G_{g,A}(\varphi_{t}(\theta^{o}))$ defines an evolution on the set of effective coupling graphs. In this section, we briefly consider possible dynamics of these effective coupling graphs.

Suppose that $H\in H_{N}$ is an effective coupling graph realized for (1.1) with coupling function g for some *θ*.

**Definition 4.1.**

The graph ***H*** can be *stably realized* if there is an asymptotically stable invariant open set *B* such that *B*⊂*Θ*_g, ***A***_(***H***). Moreover, if *B* = *Θ*_g, ***A***_(***H***), then we say that the graph ***H*** is *completely stably realized*.

In other words, for a stably realized effective coupling graph ***H***, there is an open set of *θ*^*o*^ such that

4.1 $$G_{g,A}(\varphi_{t}(\theta^{o}))=H$$

for large enough *t*. If ***H*** is completely stably realized then this holds for all *θ*^*o*^∈*Θ*_g, ***A***_(***H***).

By constructing a coupling function with a stable (relative) equilibrium, we now strengthen proposition 3.4 to show that for any ‘sufficiently connected’ ***H*** there exists a g such that the effective coupling graph ***H*** can be stably realized. To prove this result, recall the following spectral graph property [30,54].

**Proposition 4.2 ([54, Corollary 1]).**

Let ***H*** be a graph admitting a spanning diverging tree. Consider the Laplacian matrix *L*^***H***^ with coefficients

$$L_{jk}^{H}=\left\{ \begin{array}{ll} -A_{jk}^{H} & \text{if }j\neq k,\\ \sum_{\ell =1,\ell \neq k}^{N}A_{\ell k}^{H} & \text{if }k=j, \end{array}\right.$$

where *A*^***H***^ denotes the adjacency matrix of the graph ***H***. Then the multiplicity of the eigenvalue 0 in the spectrum of *L*^***H***^ is one.

We use this to prove the following result.

**Proposition 4.3.**

*For any* $H\in H_{N}$ *admitting a spanning diverging tree*, *there is a coupling function* g *such that the oscillator network* (1.2) *has a locally asymptotically stable relative equilibrium* (*Ωt* + *θ*^*o*^_1_, …, *Ωt* + *θ*^*o*^_*N*_) *satisfying* $G_{g}(\theta^{o})=H$. *In other words*, *there exists a coupling function* g *that stably realizes* ***H***.

**Proof.**

Let *θ*^*o*^ be a generic point such that all the terms *θ*^*o*^_*j*_ − *θ*^*o*^_*k*_, *j*≠*k*, are distinct. The proof now proceeds in two steps; see also [47]. First, we construct a coupling function satisfying specific conditions (specified below) and show that *θ*^*o*^ is a relative equilibrium of (1.2). Second, we show that for g, the relative equilibrium *θ*^*o*^ is locally asymptotically stable. In the following, we write $1=(1,\ldots ,1)\in R^{N}$ and let *Ωt* · **1** + *θ*^*o*^ denote the point (*Ωt* + *θ*^*o*^_1_, …, *Ωt* + *θ*^*o*^_*N*_).

*Step 1.* We now construct a coupling function g that satisfies the conditions

$$\begin{array}{rl} & \text{(i)}\;G_{g}(\theta^{o})=H,\\ & \text{(ii)}\;g^{\prime }(\theta_{j}^{o}-\theta_{k}^{o})>0\;\text{for all }(j,k)\in E(H)\\ \;\text{and}\; & \text{(iii)}\;\frac{1}{N}\sum_{j=1}^{N}g(\theta_{j}^{o}-\theta_{k}^{o})=\frac{1}{N}\sum_{j=1}^{N}g(\theta_{j}^{o}-\theta_{1}^{o})\;\text{for }k=1,\ldots ,N. \end{array}$$

By assumption all *θ*^*o*^_*j*_ − *θ*^*o*^_*k*_, *j*≠*k*, are distinct and hence we can specify g and g′ in sufficiently small neighbourhoods of these points. To satisfy (i) for given ***H***, choose g such that *θ*^*o*^_*j*_ − *θ*^*o*^_*k*_∈DZ(*g*) if and only if (*j*, *k*)∉*E*(***H***). For phase differences *θ*^*o*^_*j*_ − *θ*^*o*^_*k*_∈LZ(*g*), it is easy to see that we can specify g, g′ such that conditions (ii) and (iii) are satisfied. This yields a coupling function g that satisfies all three conditions.

Note that by condition (iii), we have *Ω* such that

$$\Omega =\omega +\frac{1}{N}\sum_{j=1,j\neq k}^{N}g(\theta_{j}^{o}-\theta_{k}^{o})$$

for all of k∈{1,…,N}. This implies that *Ωt* · **1** + *θ*^*o*^ is a relative equilibrium of (1.2).

*Step 2.* Second, we linearize (1.2) at the relative equilibrium *θ*^*o*^ and check that the conditions (i),(ii),(iii) on g also ensure asymptotic stability of *θ*^*o*^. For *χ* = *θ* − (*Ωt* · **1** + *θ*^*o*^), we have

$${\dot{\chi }}_{k}={\dot{\theta }}_{k}-\Omega =\omega +\frac{1}{N}\sum_{j=1,j\neq k}^{N}g(\chi_{j}-\chi_{k}+\theta_{j}^{o}-\theta_{k}^{o})-\Omega .$$

This yields the linearized equation ${\dot{\chi }}_{k}=\frac{1}{N}\sum_{j=1,j\neq k}^{N}g^{\prime }(\theta_{j}^{o}-\theta_{k}^{o})(\chi_{j}-\chi_{k})$ at *θ*^*o*^. Therefore, by condition (ii), the linearized equation at *Ωt* · **1** + *θ*^*o*^ read

4.2 $${\dot{\chi }}_{k}=\frac{1}{N}\sum_{j=1}^{N}A_{jk}^{H}\;g^{\prime }(\theta_{j}^{o}-\theta_{k}^{o})(\chi_{j}-\chi_{k}),\;1\leq k\leq N.$$

Now set *T*_*jk*_ = *A*^***H***^_*jk*_ g′(*θ*^*o*^_*j*_ − *θ*^*o*^_*k*_) for *j*≠*k* and $T_{kk}=-\sum_{j=1}^{N}A_{jk}^{H}g^{\prime }(\theta_{j}^{o}-\theta_{k}^{o})=-\sum_{j=1}^{N}T_{jk}$ to write (4.2) in matrix form

$${\dot{\chi }}_{k}=\frac{1}{N}\sum_{j=1}^{N}T_{jk}\chi_{j}.$$

The stability conditions are given by the spectrum S(T) of the matrix *T*. In fact −*T* is a Laplacian matrix with spectrum

$$S(T)=\left\{0,\lambda_{2},\ldots ,\lambda_{N}\right\},$$

where the eigenvalue 0 corresponds to the direction **1** = (1, …, 1) along the group orbit of the phase-shift symmetry. This means that stability of *Ωt* + *θ*^*o*^ is only determined by the eigenvalues *λ*_2_, …, *λ*_*N*_.

Note that none of the eigenvalues *λ*_2_, …, *λ*_*N*_ are equal to zero, i.e. zero is a simple eigenvalue of *T*. Indeed, let $\tilde{H}$ denote the graph of which adjacency matrix $A^{\tilde{H}}$ is defined by $A_{jk}^{\tilde{H}}=T_{jk}$ for *j*≠*k*. By condition (ii) we have $A_{jk}^{\tilde{H}}=0$ if and only if *A*^***H***^_*jk*_ = 0. This means that $E(H)=E(\tilde{H})$ and therefore $\tilde{H}$ has a spanning diverging tree: by proposition 4.2 the eigenvalue 0 of the Laplacian matrix of $\tilde{H}$ (i.e. of the matrix −*T*) is simple, which means that 0 is a simple eigenvalue of *T*. We also remark that by condition (ii) and by the Gershgorin circle theorem, all the eigenvalues of S(T) belong to the discs centred in *T*_*kk*_ < 0 and of radius −*T*_*kk*_. We can thus conclude that the eigenvalues *λ*_2_, …, *λ*_*N*_ have all a negative real part and so *Ωt* · **1** + *θ*^*o*^ is an asymptotically stable relative equilibrium. ▪

In fact proposition 4.3 can be generalized to (1.1) in a similar way.

**Corollary 4.4.**

*Assume that* H∈H(A) *admits a spanning diverging tree. Then there is a coupling function* g *such that* (*1.1*) *has an asymptotically stable relative equilibrium* (*Ωt* + *θ*^*o*^_1_, …, *Ωt* + *θ*^*o*^_*N*_) *satisfying* $G_{g,A}(\theta^{o})=H$. *In other words*, *there exists a coupling function* g *that stably realizes* ***H*** *for* (*1.1*).

**Proof.**

The proof works in an exactly similar way as in proposition 4.3. Under the same conditions (i), (ii), (iii) above, the linearized equation at *Ωt* · **1** + *θ*^*o*^ reads

$${\dot{\chi }}_{k}=\frac{1}{N}\sum_{j=1}^{N}A_{jk}A_{jk}^{H}\;g^{\prime }(\theta_{j}^{o}-\theta_{k}^{o})(\chi_{j}-\chi_{k}),\;1\leq k\leq N,$$

and the coefficients of the corresponding Laplacian matrix *T* are

$$T_{jk}=A_{jk}A_{jk}^{H}g^{\prime }(\theta_{j}^{o}-\theta_{k}^{o})\;\text{for }j\neq k$$

and

$$T_{kk}=-\sum_{j=1}^{N}A_{jk}A_{jk}^{H}\;g^{\prime }(\theta_{j}^{o}-\theta_{k}^{o})=-\sum_{j=1}^{N}T_{jk}.$$

Denoting again by $\tilde{H}$ the graph with adjacency matrix $A_{jk}^{\tilde{H}}=T_{jk}$ for *j*≠*k*, we have that $A_{jk}^{\tilde{H}}=0$ if and only if *A*^***H***^_*jk*_ = 0 by the assumptions made for ***H***. Thus, $\tilde{H}$ admits a spanning diverging tree. As in the proof of proposition 4.3, this gives again the asymptotic stability of the relative equilibrium (*Ωt* + *θ*^*o*^_1_, …, *Ωt* + *θ*^*o*^_*N*_). □

We finish with a brief discussion of a sufficient condition for an effective coupling graph ***H*** to be completely stably realized. Suppose that *θ*∈int(*Θ*_g, ***A***_(***H***)) and write the coupled oscillator network (1.1) as $\dot{\theta }=F(\theta )$. Suppose that the boundary ∂*Θ*_g, ***A***_(***H***) is a locally *N* − 1-dimensional semialgebraic set; this is typically the case as discussed in §2b. Let ***n***(*θ*) denote the piecewise defined normal vector pointing into the interior. One can now obtain sufficient conditions for ***H*** being invariant by imposing that 〈***n***(*θ*), *F*(*θ*)〉 > 0 for almost all *θ*∈∂*Θ*_g, ***A***_(***H***).

## 5. Effective coupling graphs for networks of two and three oscillators

For coupling functions with an arbitrary number of dead zones, all effective coupling graphs can be realized as outlined above. But what is the global picture of the dynamics for the minimal case of a coupling function with a single dead zone? In this section, we concentrate on this question by exploring small all-to-all coupled phase oscillator networks (1.2) with a coupling function g∈F(1). First, we briefly consider a network of *N* = 2 oscillators where there are only four possible effective coupling graphs. Then we paint the picture for *N* = 3 oscillators; there are 64 possible effective coupling graphs and we explore the dynamics numerically.

### (a) Networks of two oscillators

One can easily demonstrate that a single dead zone and a single live zone is sufficient to realize all effective coupling graphs for (1.2) with *N* = 2 oscillators. More precisely, choose any g∈F(1) with LZ(g) = [ − *a*, 2*a*] for *a* < *π*/2, where all inequalities are understood in the interval [ − *π*, *π*]. Then

$$G_{g}(0,c)=\left\{ \begin{array}{ll} K_{2} & \text{if }c\in (-a,a),\\ P_{1,2} & \text{if }c\in (a,2a),\\ P_{2,1} & \text{if }c\in (-2a,-a),\\ \emptyset_{2} & \text{if }c\in (-\pi ,-2a)\cup (2a,\pi ). \end{array}\right.$$

This shows that there is a single coupling function that realizes all four subgraphs of ***K***_2_. Note that if g is dead zone symmetric then only the undirected graph ***K***_2_ and **∅**_2_ can be realized (cf. proposition 3.7(iii)).

### (b) Networks of three oscillators

**Figure 4.**
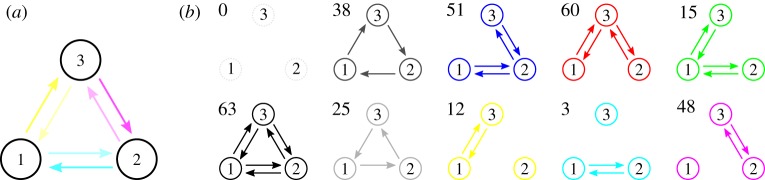
The colour scheme to identify the graphs in $H_{3}$. (*a*) The shades of cyan, magenta and yellow identified with each directed edge of ***K***_3_. If multiple edges are present, the colours are added. Examples of graphs $H\in H_{3}$ in their associated colours, as well as the corresponding graph numbers *ν*(***H***), as defined in (5.1), are shown in (*b*). The subgraphs where all edges to/from a given node are present (and no others) are associated with the colours red, green and blue. The symmetry that permutes the three nodes acts on the colour scheme by permuting the colour channels. Hence, graphs which are invariant under this symmetry operation have a colour that is invariant under permutation of the colour channels; this includes white for **∅**_3_, black for ***K***_3_, and shades of grey for the directed cycles ***C***_1,2,3_, ***C***_3,2,1_. (Online version in colour.)

We now consider all-to-all coupled networks of *N* = 3 oscillators. Since ***K***_3_ has six edges, there are 2^6^ = 64 different possible effective coupling graphs. By assigning a colour to each edge, we create a scheme that assigns a unique colour to each graph and permutations of the three nodes correspond to permutations of the colour channels. This assignment is outlined in figure 4 together with examples of graphs coloured in their respective colour. Moreover, the possible effective coupling graphs can be numbered according to the edges that are present. More specifically, let $H\in H_{3}$ and write *A*^***H***^ for the associated 3 × 3 adjacency matrix. Define the *graph number*

5.1 $$\nu (H)=A_{12}^{H}+2A_{21}^{H}+4A_{13}^{H}+8A_{31}^{H}+16A_{23}^{H}+32A_{32}^{H}\in \left\{0,\ldots ,63\right\},$$

which uniquely encodes the realized effective coupling graph as an integer. In particular, we have *ν*(**∅**_3_) = 0 and *ν*(***K***_3_) = 63; more examples are given in figure 4.

**Figure 5.**
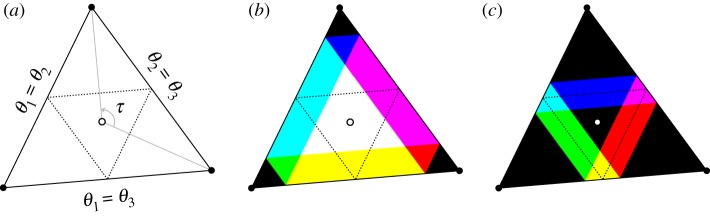
The sets *Θ*_g_(***H***) for different ***H*** partition the canonical invariant region C for the fully symmetric system of *N* = 3 oscillators. The CIR is sketched in (*a*): its boundary is given by the sets *θ*_1_ − *θ*_2_ = 0, *θ*_2_ − *θ*_3_ = 0 and *θ*_3_ − *θ*_1_ = 0 (black lines) which intersect in *Θ*^sync^ (black dot, •). The splay phase *Θ*^splay^ is the centroid (hollow dot, °) and is the fixed point of the residual $Z_{3}=\langle \tau \rangle$ symmetry which rotates the CIR (indicated by grey lines). Dashed lines indicate phase configurations where one phase difference is equal to *π*. For a dead zone symmetric coupling function g∈F(1) only the undirected subgraphs of ***K***_3_ can be realized; these correspond to the ones shown in figure 4 excluding the cycles. (*b*) The partition of the CIR for DZ(g) = (*π*/3, 5*π*/3). (*c*) The partition for a dead zone symmetric coupling function with DZ(g) = (5*π*/6, 7*π*/6). (Online version in colour.)

**Figure 6.**
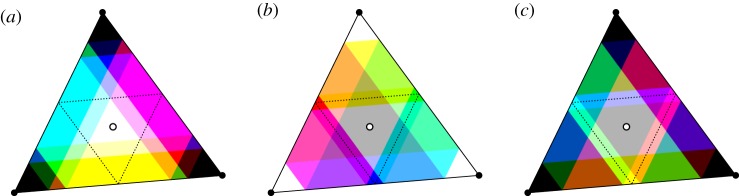
Many effective coupling graphs are possible for *N* = 3 oscillators and a general coupling function g∈F(1) with one dead zone. As in figure 5, the sets *Θ*_g_(***H***) are plotted in the colour corresponding to the effective coupling graph ***H*** in the colour scheme of figure 4. We have DZ(g) = (*π*/3, 3*π*/2) in (*a*), DZ(g) = ( − (*π*/3), 11*π*/12) in (*b*) and DZ(g) = (*π*/3, 11*π*/12) in (*c*).

For a given coupling function g∈F(1), the sets *Θ*_g_(***H***), $H\in H_{3}$, partition the CIR C. To visualize which region of phase space is associated with a given effective coupling graph, we colour the part of C accordingly. If g is dead zone symmetric, the dead zone can be parametrized by a single parameter *a*∈(0, *π*) that represents the beginning (or end) of the dead zone. Now suppose that 0∈LZ(g) so $G_{g}(\Theta^{\mathrm{sync}})=K_{3}$—the case 0∈DZ(g) is analogous by ‘inverting’ edges (or colours). There are two qualitatively different cases that are shown in figure 5: if 0 < *a* < 2*π*/3 then $G_{g}(\Theta^{\mathrm{splay}})=\emptyset_{3}$ and if 2*π*/3 < *a* < *π* then $G_{g}(\Theta^{\mathrm{splay}})=K_{3}$. Many more cases are possible for a general function g∈F(1); rather than paint a complete picture, we illustrate some cases in figure 6.

The previous considerations were purely in terms of the structure of the effective coupling graph. We now look at examples of the system's dynamics and explore how the effective coupling graph changes along trajectories. To this end, we examine the dynamics of (1.2) with *N* = 3 and the coupling function

5.2 $$g(\psi )=-\sin(\psi +\alpha )h(\psi )\;\text{where }h(\psi )=\frac{1}{2}(\tanh(\varepsilon^{-1}(\cos b-\cos(a-\psi ))+1)$$

for constants *a*∈[0, 2*π*), *b*∈[0, *π*), *ε* > 0 and *α*∈[0, 2*π*). This coupling function is a modulated Kuramoto–Sakaguchi coupling with phase-shift parameter *α*. We call

5.3 $${\text{DZ}}^{\varepsilon }(g)=\left\{\theta \;|\;\left|\theta -a\right|<b\right\}$$

the approximate dead zone of the coupling function (5.2) since in the limit ε→0 the coupling function (5.2) has a single dead zone DZ(g)={θ | |θ−a|<b} centred at *a* of half-width *b*; here the inequality is to be understood modulo 2*π*. In the following, we fix *ε* = 5 × 10^−3^ and *α* = 1.3.

**Figure 7.**
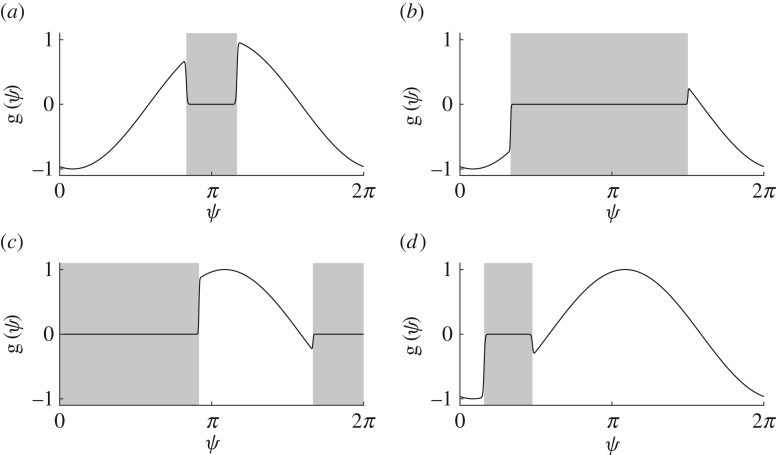
The coupling functions (5.2) provide examples of coupling functions g∈F(1) with one dead zone; here *ε* = 5 × 10^−3^ and *α* = 1.3. The shaded area indicates the dead zone of the coupling function. (*a*) We have a dead zone symmetric coupling function with DZ^*ε*^(g) = (5*π*/6, 7*π*/6); cf. figure 5*b*. (*b*) We have DZ^*ε*^(g) = (*π*/3, 3*π*/2); cf. figure 6*a*. (*c*) We have DZ^*ε*^(g) = ( − (*π*/3), 11*π*/12); cf. figure 6*b*. (*d*) We have DZ^*ε*^(g) = (0.5, 1.5).

**Figure 8.**
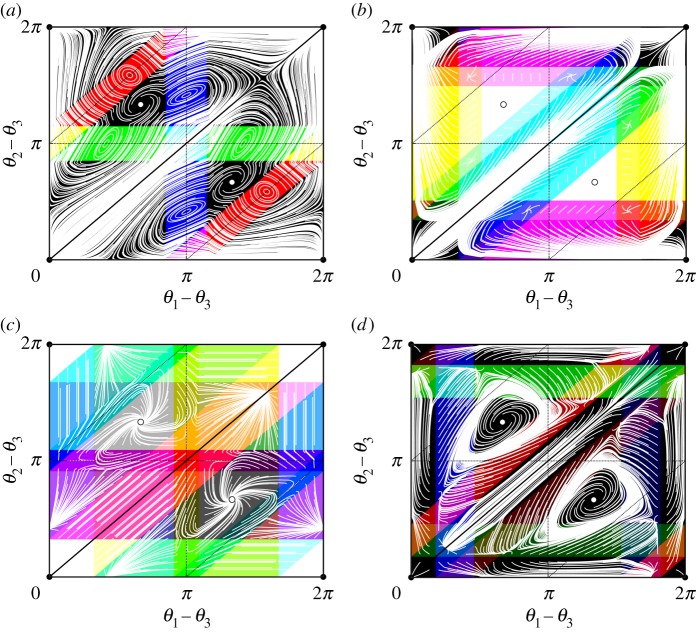
The phase space for (1.2) with *N* = 3 oscillators and coupling function g with one dead zone as in (5.2) and parameters as in figure 7*a*–*d* respectively. As in figures 5 and 6, black lines indicate the sets *θ*_1_ − *θ*_2_ = 0, *θ*_2_ − *θ*_3_ = 0 and *θ*_3_ − *θ*_1_ = 0 which intersect in *Θ*^sync^ (black dot, •) that bound C and its symmetric image. The splay phases are indicated by hollow dots (°) and dashed lines indicate phase configurations where one phase difference is equal to *π*. As above, the colouring indicates the effective coupling graph overlaid by trajectories started on a regular grid, shown in white—a very wide range of effective coupling graphs are realised; see figure 9. For (*b*) and (*c*), there are white regions of trivial dynamics where no trajectories are present: these correspond to the effective coupling graph **∅**_3_. Finally, note that for (*a*) and (*c*) there are trajectories that visit *Θ*_g_(***H***) for multiple $H\in H_{3}$ as time evolves. (Online version in colour.)

We explore the dynamics for four examples of coupling functions (5.2) with approximate dead zones shown in figure 7. Recall that without dead zones (and for any *N*), the dynamics for Kuramoto–Sakaguchi coupling is well known: depending on the parameter *α*, either full synchrony *Θ*^sync^ or an anti-phase configuration is stable; see also [55]. Now for each of the Kuramoto–Sakaguchi coupling functions with a dead zone, figure 8 shows a partition of phase space by effective coupling graph (using the same colour scheme as in figures 5 and 6) together with a phase portrait for trajectories of (1.2) integrated forwards from a grid of initial conditions. Note that the dynamics in *Θ*_g_(**∅**_3_)—coloured in white—are trivial and we find, for example, periodic trajectories that visit *Θ*_g_(***H***) for multiple $H\in H_{3}$ as time evolves. Such dynamics are impossible for Kuramoto–Sakaguchi coupling without dead zones.

**Figure 9.**
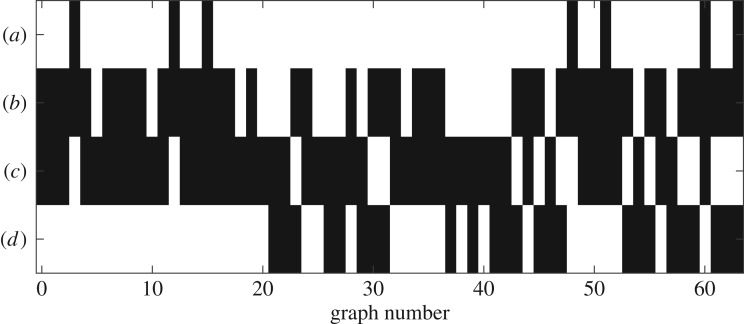
The possible effective coupling graphs realized using *N* = 3 and (5.2) for parameters as in figure 7 and some *θ*. Black indicates *Θ*_g_(***H***)≠∅ for $H\in H_{3}$ with a given graph number, and white indicates *Θ*_g_(***H***) = ∅. Since (*a*) is a dead zone symmetric coupling function, only undirected subgraphs are realized. By contrast, taking all effective coupling graphs that are realized by coupling functions (*b*) and (*c*) together, one obtains all possible subgraphs of ***K***_3_.

Finally, we consider the entirety of the effective interaction graphs that are realized by a given coupling function. Figure 9 shows the set of realized effective coupling graphs corresponding to the coupling functions in figure 7 sorted by their graph numbers. We are unable to find a single coupling function g with one live zone that can realize all possible effective coupling graphs—however, we note that a combination of two coupling functions (for example, (*b*) and (*c*)) suffice to cover all cases.

## 6. Conclusion

In this paper, we have demonstrated that the *effective* coupling graph of a dynamical network is subtly related to the network structure, the system state and the presence of dead zones in the interaction. Working with coupled phase oscillator networks (1.1), we give constructions of coupling functions g that achieve any desired subnetwork, possibly using the same g (corollary 3.5), even in the special and highly symmetric case of all-to-all coupling (1.2).

In terms of structural questions, we obtain a number of conditions on g and *θ* that guarantee the presence of certain coupling structures in $G_{g}(\theta )$. There are several natural questions that relate to the number, location and lengths of the dead zones to the set of realizable effective coupling graphs. For example, the coupling functions in figure 7*b*,*c* together can realize all possible (embedded) subgraphs of ***K***_3_. Two specific questions in this direction for (1.2) are

— What is the minimum number *n* of dead zones such that there is a g∈F(n) that realizes all $H\in H_{N}$?
— What is the minimum *m* for which one can find *m* coupling functions, each with at most *n* dead zones, such that any $H\in H_{N}$ is realized by at least one of these functions?^3^

In terms of the dynamics, probably the most interesting problems relate to how the dynamics of the coupled system interacts with the effective coupling as $G_{g}(\varphi_{t}(\theta^{o}))$ changes along a trajectory starting at *θ*^*o*^. This is briefly explored in §4 and in the examples in §5, but we do not have a complete picture as yet. For example, can one determine which effective coupling graphs can be stably realized, and which can be only transiently realized? What does the passage through effective coupling graphs tell us about the underlying dynamics? How does the partition of phase space into basins of attraction map on to the partition of phase space by *Θ*_g_(***H***)?

Here, we only considered phase oscillator networks with coupling functions that have simple dead zones, i.e. there are a finite set of non-trivial intervals on which the coupling function vanishes. This could be developed in three directions: first, one may want to examine coupling with an infinite set of dead zones (though this is likely to be not of much relevance to applications). Second, one could look at the case where the coupling function is locally constant on several intervals where it takes distinct values. Third, one would like to get explicit results for coupling functions with approximate dead zones, i.e. intervals on which the coupling functions are small but non-zero. In this direction, it would be desirable to prove explicit results concerning how well (and over what time scale) networks with dead zones approximate networks where interaction between nodes is small (but non-zero) in parts of phase space.

Finally, we have restricted ourselves here to discussion of these questions for coupled phase oscillators where all interactions are governed by a single periodic phase interaction/coupling function g. On the one hand, it would be desirable to link the coupling functions considered here to nonlinear oscillator networks through a phase reduction. On the other hand, it would be interesting to explore how these results can be generalized, for example, to more general dynamical systems with pairwise coupling of the form

$${\dot{x}}_{k}=f(x_{k})+\sum_{j\neq k}g(x_{j},x_{k})$$

for $x_{k}\in R^{d}$, where *g*(*x*_*j*_, *x*_*k*_) is null in some open subset of $R^{d}\times R^{d}$. Finally, dead zones could also be present in multi-way interactions [1,4], i.e. interactions where the coupling to *x*_*k*_ depends simultaneously on the relative position of several of the *x*_*j*_ with *j*≠*k*, and not only on one of them.

## Footnotes

1 Writing the image of a subset *A* of the range of a function *F* as *F*(*A*): = {*F*(*a*):*a*∈*A*}.

2 Note that if there are *n* > 1 dead zones there must also be *n* live zones, while for *n* = 1 there can be 0 or 1 live zones, and for *n* = 0 there is necessarily one live zone.

3 Figure 9 gives evidence that *m* ≤ 2 for *N* = 3 and *n* = 1.

## Data accessibility

This article has no additional data.

## References

[1] Stankovski T, Pereira T, McClintock PVE, Stefanovska A 2017 Coupling functions: universal insights into dynamical interaction mechanisms. Rev. Mod. Phys. 89, 045001 (10.1103/RevModPhys.89.045001)

[2] Pikovsky A, Rosenblum M, Kurths J 2001 Synchronization. Cambridge Nonlinear Science Series, no. 12 Cambridge, UK: Cambridge University Press.

[3] Tanaka T, Aoyagi T 2011 Multistable attractors in a network of phase oscillators with three-body interactions. Phys. Rev. Lett. 106, 224101 (10.1103/PhysRevLett.106.224101) 21702602

[4] Ashwin P, Bick C, Rodrigues A 2016 Chaos in generically coupled phase oscillator networks with nonpairwise interactions. Chaos 26, 094814 (10.1063/1.4958928) 27781441

[5] Bick C 2018 Heteroclinic switching between chimeras. Phys. Rev. E 97, 050201(R) (10.1103/PhysRevE.97.050201) 29906933

[6] Levine JM, Bascompte J, Adler PB, Allesina S 2017 Beyond pairwise mechanisms of species coexistence in complex communities. Nature 546, 56–64. (10.1038/nature22898) 28569813

[7] Uriu K, Tei H 2019 A saturated reaction in repressor synthesis creates a daytime dead zone in circadian clocks. PLoS Comput. Biol. 15, e1006787 (10.1371/journal.pcbi.1006787) 30779745 PMC6396941

[8] Glass L, Kauffman SA 1973 The logical analysis of continuous, non-linear biochemical control networks. J. Theor. Biol. 39, 103–129. (10.1016/0022-5193(73)90208-7) 4741704

[9] Glass L, Pasternack JS 1978 Stable oscillations in mathematical models of biological control systems. J. Math. Biol. 6, 207–223. (10.1007/BF02547797)

[10] Edwards R 2000 Analysis of continuous-time switching networks. Physica D 146, 165–199. (10.1016/S0167-2789(00)00130-5)

[11] Hahnloser RHR, Seung HS 2003 Permitted and forbidden sets in linear threshold networks. Neural Comput. 15, 621–638. (10.1162/089976603321192103) 12620160

[12] Huttinga Z, Cummins B, Gedeon T, Mischaikow K 2018 Global dynamics for switching systems and their extensions by linear differential equations. Physica D 367, 19–37. (10.1016/j.physd.2017.11.003) 29867284 PMC5984053

[13] Curto C, Geneson J, Morrison K 2019 Fixed points of competitive threshold-linear networks. Neural Comput. 31, 94–155. (10.1162/neco_a_01151) 30462583

[14] Goodwin BC 1965 Oscillatory behavior in enzymatic control processes. Adv. Enzyme Regul. 5, 425–428. (10.1016/0065-2571(65)90067-1) 5861813

[15] Tyson JJ, Chen KC, Novak B 2003 Sniffers, buzzers, toggles and blinkers: dynamics of regulatory and signaling pathways in the cell. Curr. Opin Cell Biol. 15, 221–231. (10.1016/S0955-0674(03)00017-6) 12648679

[16] Poignard C, Chaves M, Gouzé J 2018 A stability result for periodic solutions of nonmonotonic smooth negative feedback systems. SIAM J. Appl. Dyn. Sys. 17, 1091–1116. (10.1137/17M1141205)

[17] Poignard C, Chaves M, Gouzé J 2016 Periodic oscillations for nonmonotonic smooth negative feedback circuits. SIAM J. Appl. Dyn. Sys. 15, 257–286. (10.1137/15M1033368)

[18] Hastings S, Tyson JJ, Webster D 1977 Existence of periodic solutions for negative feedback cellular control systems. J. Differ. Equ. 25, 39–64. (10.1016/0022-0396(77)90179-6)

[19] Tyson JJ 1975 On the existence of oscillatory solutions in negative feedback cellular control processes. J. Math. Biol. 1, 311–315. (10.1007/BF00279849)

[20] Poignard C 2014 Inducing chaos in a gene regulatory network by coupling an oscillating dynamics with a hysteresis-type one. J. Math. Biol. 69, 335–368. (10.1007/s00285-013-0703-5) 23842815

[21] Shahrear P, Glass L, Edwards R 2015 Chaotic dynamics and diffusion in a piecewise linear equation. Chaos 25, 033103 (10.1063/1.4913417) 25833425

[22] Soulé C 2003 Graphic requirements for multistationarity. Complexus 1, 123–133. (10.1159/000076100)

[23] Domijan M, Pécou E 2012 The interaction graph structure of mass-action reaction networks. J. Math. Biol. 65, 375–402. (10.1007/s00285-011-0462-0) 21858686

[24] Sjöström PJ, Rancz EA, Roth A, Häusser M 2008 Dendritic excitability and synaptic plasticity. Physiol. Rev. 88, 769–840. (10.1152/physrev.00016.2007) 18391179

[25] Deffuant G, Neau D, Amblard F, Weisbuch G 2000 Mixing beliefs among interacting agents. Adv. Comp. Sys. 03, 87–98. (10.1142/S0219525900000078)

[26] Hegselmann R, Krause U 2002 Opinion dynamics and bounded confidence: models, analysis and simulation. J. Artif. Soc. Simul. 5, 1–33.

[27] Izhikevich EM 2007 Dynamical systems in neuroscience: the geometry of excitability and bursting. Cambridge, MA: MIT Press.

[28] Ashwin P, Coombes S, Nicks R 2016 Mathematical frameworks for oscillatory network dynamics in neuroscience. J. Math. Neurosci. 6, 2 (10.1186/s13408-015-0033-6) 26739133 PMC4703605

[29] Fiedler M 1973 Algebraic connectivity of graphs. Czech. Math. J. 23, 298–305.

[30] Agaev R, Chebotarev P 2000 The matrix of maximum out forests of a digraph and its applications. Autom. Remote Control 61, 1424–1450.

[31] Pecora LM, Carroll TL 1998 Master stability functions for synchronized coupled systems. Phys. Rev. Lett. 80, 2109–2112. (10.1103/PhysRevLett.80.2109)

[32] Barahona M, Pecora LM 2002 Synchronization in small-world systems. Phys. Rev. Lett. 89, 054101 (10.1103/PhysRevLett.89.054101) 12144443

[33] Wu CW, Chua LO 1996 On a conjecture regarding the synchronization in an array of linearly coupled dynamical systems. IEEE Trans. Circuits Syst. I 43, 161–165. (10.1109/81.486440)

[34] Belykh I, Hasler M, Lauret M, Nijmeijer H 2005 Synchronization and graph topology. Int. J. Bif. Chaos 15, 3423–3433. (10.1142/S0218127405014143)

[35] Nishikawa T, Motter AE 2006 Synchronization is optimal in non-diagonalizable networks. Phys. Rev. E 73, 065106 (10.1103/PhysRevE.73.065106) 16906895

[36] Motter TNAE, Lai YC, Hoppensteadt FC 2003 Heterogeneity in oscillator networks: are smaller worlds easier to synchronize? Phys. Rev. Lett. 91, 014101 (10.1103/PhysRevLett.91.231101) 12906539

[37] Li C, Sun W, Kurths J 2007 Synchronization between two coupled complex networks. Phys. Rev. E 76, 046204 (10.1103/PhysRevE.76.046204) 17995079

[38] Pereira T, Eldering J, Rasmussen M, Veneziani A 2014 Towards a theory for diffusive coupling functions allowing persistent synchronization. Nonlinearity 27, 501–525. (10.1088/0951-7715/27/3/501)

[39] Pogromsky A, Nijmeijer H 2001 Cooperative oscillatory behavior of mutually coupled dynamical systems. IEEE Trans. Circ. Sys. I: Fundam. Theory Appl. 48, 152–162. (10.1109/81.904879)

[40] Milanese A, Sun J, Nishikawa T 2010 Approximating spectral impact of structural perturbations in large networks. Phys. Rev. E 81, 046112 (10.1103/PhysRevE.81.046112) 20481791

[41] Nishikawa T, Sun J, Motter AE 2017 Sensitive dependence of optimal network dynamics on network structure. Phys. Rev. X 7, 041044 (10.1103/physrevx.7.041044)

[42] Poignard C, Pereira T, Pade J 2018 Spectra of Laplacian matrices of weighted graphs: structural genericity properties. SIAM J. Appl. Math. 78, 372–394. (10.1137/17M1124474)

[43] Poignard C, Pade J, Pereira T 2019 The effects of structural perturbations on the synchronizability of diffusive networks. J. Nonlinear Sci. 29, 1919–1942. (10.1007/s00332-019-09534-7)

[44] Bick C, Field MJ 2017 Asynchronous networks and event driven dynamics. Nonlinearity 30, 558–594. (10.1088/1361-6544/aa4f62)

[45] Bick C, Field MJ 2017 Asynchronous networks: modularization of dynamics theorem. Nonlinearity 30, 595–621. (10.1088/1361-6544/aa4f4d)

[46] Acebrón JA, Bonilla LL, Vicente CJP, Ritort F, Spigler R 2005 The Kuramoto model: a simple paradigm for synchronization phenomena. Rev. Mod. Phys. 77, 137–185. (10.1103/RevModPhys.77.137)

[47] Orosz G, Moehlis J, Ashwin P 2009 Designing the dynamics of globally coupled oscillators. Prog. Theor. Phys. 122, 611–630. (10.1143/PTP.122.611)

[48] Ashwin P, Orosz G, Wordsworth J, Townley S 2007 Dynamics on networks of cluster states for globally coupled phase oscillators. SIAM J. Appl. Dyn. Sys. 6, 728 (10.1137/070683969)

[49] Bick C, Timme M, Paulikat D, Rathlev D, Ashwin P 2011 Chaos in symmetric phase oscillator networks. Phys. Rev. Lett. 107, 244101 (10.1103/PhysRevLett.107.244101) 22243002

[50] Ashwin P, Swift JW 1992 The dynamics of *n* weakly coupled identical oscillators. J. Nonlinear Sci. 2, 69–108. (10.1007/BF02429852)

[51] Ashwin P, Bick C, Burylko O 2016 Identical phase oscillator networks: bifurcations, symmetry and reversibility for generalized coupling. Front. Appl. Math. Stat. 2, 1–16. (10.3389/fams.2016.00007)

[52] Pikovsky A, Rosenblum M 2015 Dynamics of globally coupled oscillators: progress and perspectives. Chaos 25, 097616 (10.1063/1.4922971) 26428569

[53] Diestel R 2017 Graph theory, 5th edn Graduate Texts in Mathematics, vol. 173 Berlin, Heidelberg: Springer Berlin Heidelberg (10.1007/978-3-662-53622-3)

[54] Agaev R, Chebotarev P 2009 Coordination in multiagent systems and Laplacian spectra of digraphs. Autom. Remote Control 70, 469–483. (10.1134/S0005117909030126)

[55] Watanabe S, Strogatz SH 1994 Constants of motion for superconducting Josephson arrays. Physica D 74, 197–253. (10.1016/0167-2789(94)90196-1)

